# Association between metformin and physical activity with glucose control in adults with type 2 diabetes

**DOI:** 10.1002/edm2.206

**Published:** 2020-12-15

**Authors:** Diana Abdalhk, Michael C. Riddell, Sarah Swayze, Jennifer L. Kuk

**Affiliations:** ^1^ School of Kinesiology and Health Science York University Toronto Ontario Canada

## Abstract

**Objective:**

To examine the combined association between metformin use and physical activity on HbA1c in adults with type 2 diabetes.

**Research Design and Methods:**

Adults with type 2 diabetes from NHANES continuous survey (1999‐2018, n = 6447) were classified as active and inactive based on self‐reported engagement in moderate‐to‐vigorous or vigorous physical activity (MVPA or VigPA) and metformin use over the last month.

**Results:**

There was a significant negative main effect of metformin usage on HbA1c levels, independent of whether individuals engaged in modest levels of MVPA or VigPA. Moreover, there was a higher prevalence of metformin users with a HbA1c < 6.5% than non‐metformin users with no differences by activity status (36.1%‐39.5% versus 24.9%‐29.7%, respectively). There was a significantly lower HbA1c level (*P* = .007) and trend for a higher odds of having a HbA1c that achieved the clinical target of <7% (OR, 95% CI = 1.2, 1.0‐1.4, *P* = .06) in the MVPA than non‐MVPA group for only those not using metformin. For those using metformin, there was no difference in HbA1c levels by either MVPA or VigPA (both *P* > .05).

**Conclusions:**

There appears to be independent benefits of metformin and regular physical activity on glucose control, but the impact of these two treatments are not necessarily additive. Based on this analyses, the benefit of physical activity on HbA1c levels in type 2 diabetes is likely more apparent in those not taking metformin, as compared to those who are.

## Graphical abstract

There appears to be independent benefits of metformin and regular physical activity on glucose control, but the impact of these two treatments are not necessarily additive. Based on these analyses, the benefit of physical activity on HbA1c levels in type 2 diabetes is likely more apparent in those not taking metformin, as compared to those who are.

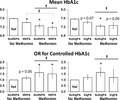

## 1 INTRODUCTION

Pharmaceutical treatment along with lifestyle management is important elements of the treatment for type 2 diabetes. Over the years, metformin has increasingly been prescribed as a first‐line pharmaceutical approach for glycaemic management in patients with type 2 diabetes.^1^ A recent estimate suggests that ~45% of people who take type 2 diabetes medications in the United States use metformin.^1^ Metformin has been shown to effectively reduce fasting and postprandial blood glucose (FPG) and glycosylated haemoglobin (HbA1c).^2^ The potent glucose lowering effect of metformin is complex and is likely achieved through its multi‐faceted actions on peripheral tissues, including liver, pancreas, muscles, fat and intestine.^3^ Earlier evidence that metformin primarily improves glycaemic control in diabetes by reducing gluconeogenesis and hepatic glucose output^4^ is being challenged by more recent work showing that the oral agent may favourably improve glycaemia through beneficial changes to the incretin profile.^5^ Metformin is also associated with increases in glucose clearance via the gut and other nonhepatic tissues.^6^, ^7^, ^8^ Similarly, exercise is associated with enhanced skeletal muscle glucose uptake, by inducing contraction‐mediated glucose transporter four (GLUT4) translocation^9^ and with improvements in liver insulin sensitivity that can reduce hepatic glucose production postprandially.^10^ The combined association between metformin and regular physical activity on glycaemic control in type 2 diabetes has been studied in a few smaller studies^11^, ^12^, ^13^, ^14^, ^15^ and some suggest that there may be acute effects of exercise on glucose,^11^, ^13^ but it is unclear whether these differences translate into meaningful chronic differences in glucose or glucose control. Some of these studies suggest that beneficial effects of exercise on glucose metabolism and insulin sensitivity may be attenuated in those taking metformin.^11^, ^13^, ^15^, ^16^ Thus, the objective of the current study is to examine the combined association between metformin and regular physical activity on glycaemic control in adults with type 2 diabetes. This work may help to elucidate whether metformin and physical activity improve glycaemia through similar or independent pathways.

## 2 METHODS AND PROCEDURES

Data were obtained from participants in the National Health and Nutrition Examination continuous surveys (NHANES 1999‐2018) in the United States. Data collection methods were approved by the National Centre for Health Statistics (NCHS) and Centers for Disease Control and Prevention (CDC).^17^ All participants signed an informed consent prior to participation in the study and ethical approval was received from the National Center for Health Statistics Institutional Review Board. Public‐use data files were used and thus these analyses did not require further ethical review from York University's Research Ethics board. The samples from continuous survey cycles were collected using a multistage stratified probability cluster design and weighted to be nationally representative of all non‐institutionalized citizens in the United States according to NHANES analytic guidelines.^18^

The analytical sample included all nonpregnant adults (≥20‐year‐old) with type 2 diabetes and complete data for medication use, physical activity, family income to poverty ratio, body mass index and HbA1c (n = 6447). Participants considered to have type 2 diabetes if they had a fasting plasma glucose of 7.0 mmol/L, were taking any anti‐hyperglycaemic medications, had an HbA1c of ≥6.5% (48 mmol/mol) or had a 2 hour oral glucose tolerance test of 11.0 mmol/L (only available 2005 and onwards). HbA1c was categorized into controlled HbA1c of <7% (Low HbA1c) and uncontrolled HbA1c of ≥7% (High HbA1c). Metformin and other medication use over the last 30 days was reported by participants and when possible, the medication containers were shown to the interviewers Prescription Medications.^19^

Laboratory analyses were conducted at the University of Missouri at Columbia and the detailed methods are published.^20^ HbA1c was obtained from blood samples via venipuncture and the percentage of HbA1c was calculated in whole blood specimens using Tosoh Automated Glycosylated Hemoglobin Analyzer HLC‐723G8 (Bio‐Rad Laboratories, Hercules, CA 1990). Fasting plasma glucose levels were measured by a hexokinase enzymatic reference method (Roche Cobas Mira, Indianapolis, IN 1988).

Physical activity in NHANES continuous surveys 1999‐2018 was assessed during a household interview by trained interviewers. Individuals were asked if they participated in any moderate‐to‐vigorous physical activity over the past 30 days. Moderate‐intensity activities are activities that cause small increases in breathing or heart rate such as brisk walking or carrying light loads for at least 10 minutes continuously. Vigorous‐intensity activities were identified as work or recreational activities that cause large increases in breathing or heart rate for at least 10 minutes continuously.^21^ Participants were classified as active if they reported engaging in any moderate and/or vigorous physical activity in the last month (MVPA and VigPA) or inactive if they did not (noMVPA and noVigPA).

Participant characteristics are reported as means (SE) or prevalence (SE) according to metformin use and physical activity status. Differences in participant characteristics were assessed using ANOVA with Bonferroni adjustment or chi‐square tests. Least squared adjusted means for HbA1c and fasting glucose were produced for Metformin‐PA groups using general linear models adjusting for age, sex, ethnicity, other type 2 diabetes medications, obesity and family poverty income ratio. Logistic regression was performed to examine the odds of having prevalent controlled HbA1c or glucose for each Metformin‐PA group, adjusting for age, sex, ethnicity, other type 2 diabetes medications, obesity and family poverty income ratio. The referent group was No Metformin use‐No PA for all models. All statistical analyses were conducted using SAS vs. 9.4 and were weighted to be representative of the US population according the NHANES analytical guidelines.^22^ Results were considered significant at *P* < .05.

## 3 RESULTS

Characteristics of participants are presented in Table 1 by physical activity status and metformin use status. Within the sample, 22% of individuals reported engaging in vigorous PA at least once per month while 48% reported engaging in MVPA. In general, participants who were active were slightly younger, more likely to be male, had a lower BMI and higher income level than inactive participants. Metformin users tended to be older and were more likely to be taking T2D medications other than metformin than those not using metformin.

**Table 1:** Participant characteristics stratified by physical activity and metformin use

Variable	No Metformin		Metformin	
	NoMVPA	MVPA	NoMVPA	MVPA
N	3147	2378	453	469
Age, y	61.3 (0.3)	57.4 (0.4)*	57.5 (0.8)^†^	54.6 (0.9)^†^
Sex				
%Male	21.0 (0.8)	24.7 (0.9)*	3.4 (0.4)	3.9 (0.3)
%Female	24.0 (0.7)	15.9 (0.6)	3.4 (0.3)	3.7 (0.3)
%White Ethnicity	63.6 (1.7)	64.2 (1.7)	57.6 (3.9)	57.8 (3.5)
BMI, kg/m^2^	33.4 (0.2)	32.2 (0.2)*	34.3 (0.6)	33.1 (0.4)
HbA1c	7.5 (0.1)	7.3 (0.1)	7.3 (0.1)	7.3 (0.1)
Glucose, mmol/L	8.5 (0.1)	8.5 (0.1)	8.8 (0.3)	9.1 (0.3)
Other T2D medications (%)	41.9 (1.3)	38.2 (1.4)	56.4 (4.1)^†^	57.6 (4.3)^†^
Family PIR	2.5 (0.1)	3.0 (0.1)*	2.5 (0.1)	2.9 (0.1)*

Variable	No Metformin		Metformin	
	NoVigPA	VigPA	NoVigPA	VigPA
N	4489	1036	721	201
Age, y	61.2 (0.3)	53.0 (0.6)*	56.9 (0.8)^†^	52.9 (1.3)*
Sex				
%Male	13.3 (0.8)	32.4 (0.8)*	2.0 (0.3)^†^	5.3 (0.4)
%Female	5.5 (0.4)	34.4 (0.8)	1.5 (0.2)	5.6 (0.4)
%White Ethnicity	64.5 (1.5)	61.8 (2.6)	57.6 (3.0)	58.2 (5.5)
BMI, kg/m^2^	33.1 (0.2)	32.0 (0.3)*	34.0 (0.5)	32.8 (0.6)
HbA1c	7.4 (0.1)	7.4 (0.1)	7.3 (0.1)	7.3 (0.2)
Glucose, mmol/L	8.5 (0.1)	8.6 (0.2)	8.9 (0.3)	9.2 (0.4)
Other T2D medications (%)	42.7 (1.1)	31.1 (2.1)*	59.9 (3.0)^†^	48.3 (5.6)^†^
Family PIR	2.7 (0.1)	3.0 (0.1)*	2.6 (0.1)	2.8 (0.1)

* Significant difference between PA groups within Metformin group (*P* < .05).

^†^ Significant difference between Metformin groups within PA group (*P* < .05).

**Figure 1:**
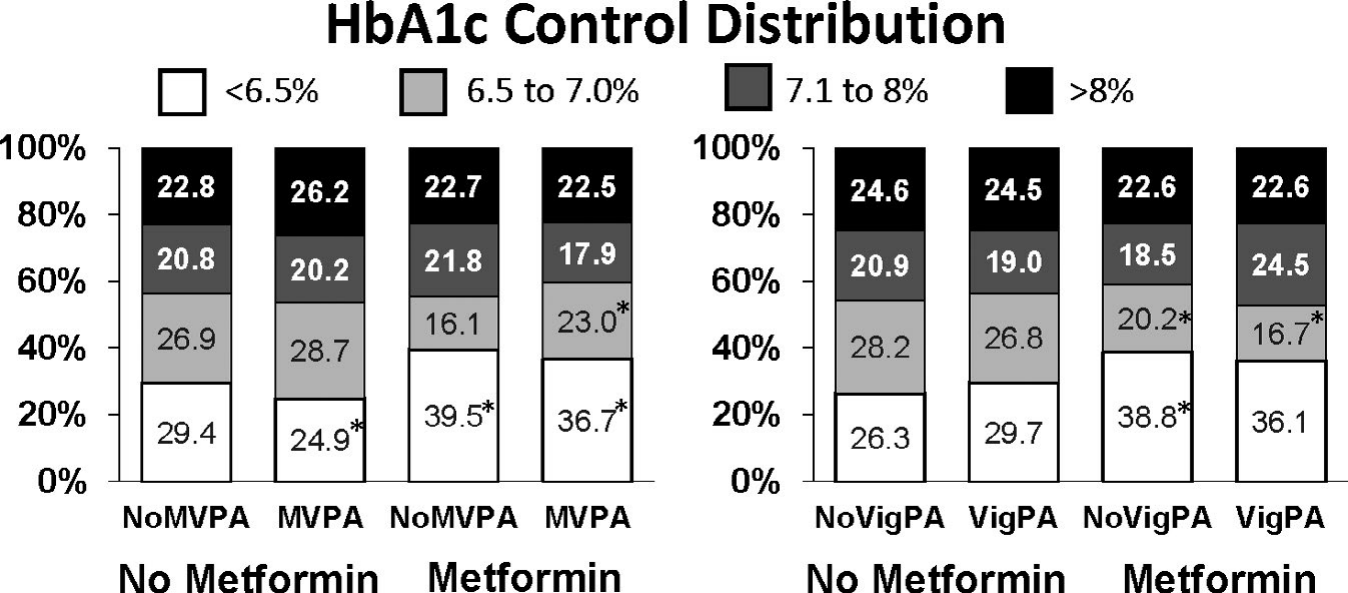
Prevalence of HbA1c Control Rates by Metformin and Physical Activity Status. *Difference from No Metformin‐no PA group (*P* < .05). MVPA, Moderate‐Vigorous Physical Activity; VigPA, Vigorous Physical Activity

The proportion of individuals in various HbA1c categories (<6.5%; 6.5 to 7%; 7.1 to 8% and > 8%) by metformin and activity status is shown in Figure 1. Individuals using metformin were more likely to attain a HbA1c of < 6.5% than non‐metformin users (*P* < .05), but no differences were observed between metformin users and non‐users in the higher HbA1c categories (HbA1c > 7.1%).

**Figure 2:**
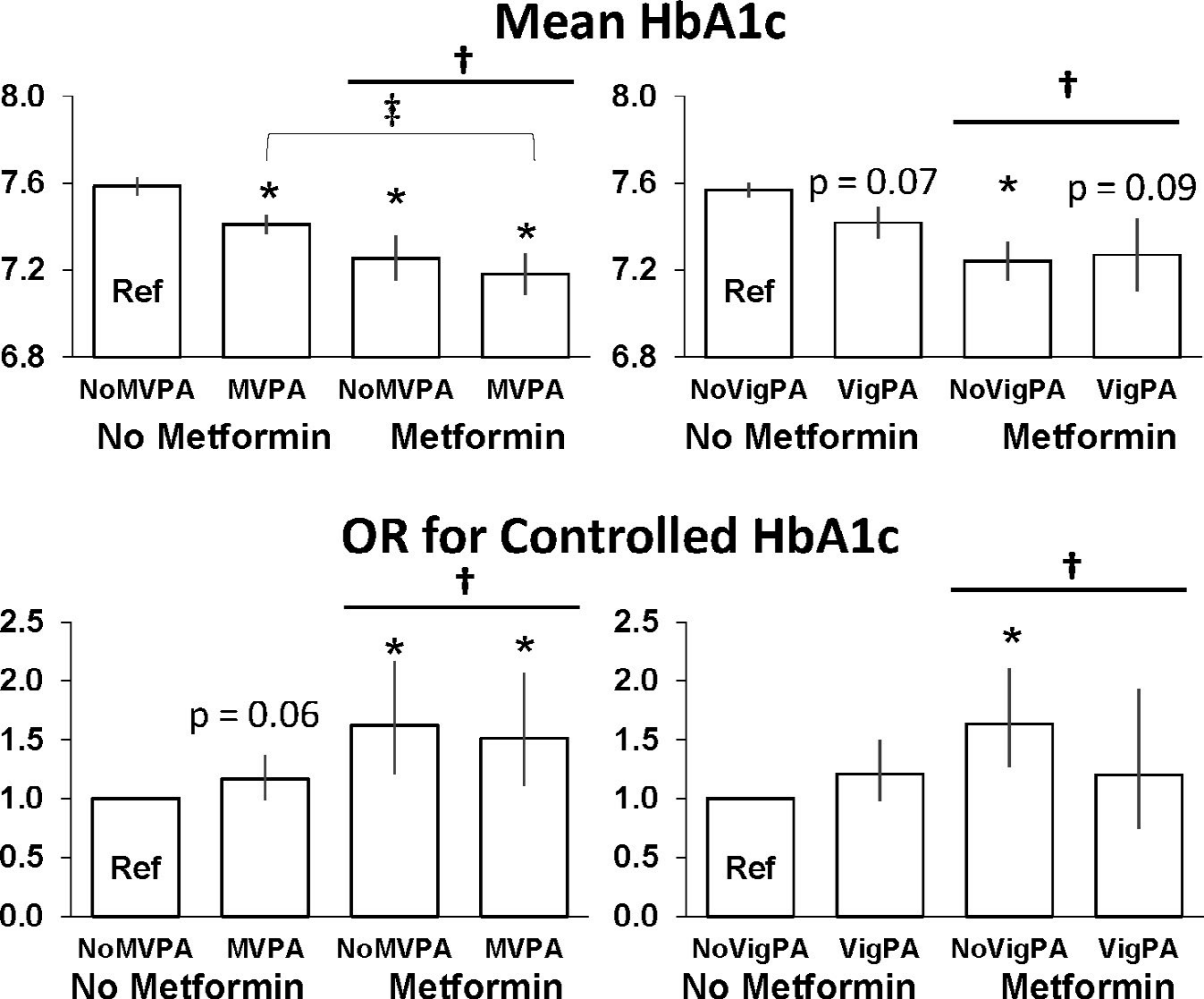
Least squared adjusted means for HbA1c and Odds Ratio (OR) for Controlled HbA1c by Metformin and Physical Activity Status. *Difference from No Metformin‐no PA group (*P* < .05). Controlled HbA1c is <7%. ^†^Significant main effect of Metformin (*P* < .05). ^‡^Significant main effect of physical activity (*P* < .05). MVPA, Moderate‐Vigorous Physical Activity; VigPA, Vigorous Physical Activity

The mean HbA1c by metformin and physical activity status adjusted for age, sex, white ethnicity, family income, other type 2 diabetes medication and obesity status use are presented in Figure 2. For HbA1c, there was an activity level by metformin use interaction (*P* < .0001), in that there was a significantly lower mean HbA1c in the MVPA group than the non‐MVPA group, but only for those not using metformin (*P* = .007). There was a significant negative main effect of metformin on HbA1c independent of MVPA and VigPA (*P* < .0001).

There was no interaction of MVPA or VigPA status and metformin use on the odds of achieving the clinically relevant HbA1c target of <7% (Figure 2, *P* > .05). However, there was a significant positive main effect of metformin use (*P* < .001), but not MVPA or VigPA (*P* > .05) on the odds of achieving a HbA1c < 7%. There was a trend for MVPA to be associated with a greater odds of achieving a HbA1c < 7%, in those not taking metformin (*P* = .06), while both metformin groups were significantly more likely to have controlled HbA1c (*P* < .01).

## 4 DISCUSSION

To our knowledge, this is the first study to determine the combined associations between metformin usage and various exercise intensity physical activity status measurements on glucose control, as measured by HbA1c level, in adults with type 2 diabetes. This study suggests that there may be independent benefits of metformin and physical activity levels on glucose control, with the benefits of physical activity being more apparent in those not taking metformin.

Several pharmaceutical randomized control trials show evidence of the effectiveness of metformin on glycaemia via reducing hepatic glucose production.^8^ However, it is clear that metformin has additional effects on glycaemic control that are not yet fully understood. The benefits of exercise on insulin sensitivity are also well described,^23^ with a meta‐analysis suggesting a 0.6% reduction of HbA1c with exercise in patients with type 2 diabetes.^24^ However, whether the benefits of exercise or physical activity are altered in patients using metformin are unclear. Consistent with some previous literature,^11^, ^16^ this study demonstrates that the glycaemic benefits of physical activity are likely attenuated in those individuals living with type 2 diabetes and already using metformin. This may indicate a basement effect wherein the beneficial effects of metformin on chronic glycaemia are not further improved by physical activity. This may suggest a common pathway for metformin and physical activity on improving glycaemia in those with type 2 diabetes. Thus, clinicians perhaps should not expect an additive benefit of physical activity on glycaemic control for their patients already using metformin. Alternatively, the lack of difference in glycaemic control with physical activity in those using metformin may also indicate that higher amounts of physical activity may be needed to elicit beneficial glycaemic effects in those using metformin. Currently, most of the literature has focused on the glycaemic effects of traditional continuous moderate‐intensity exercise and the glycaemic effect other forms of activity such as high intensity and/or interval training are less clear, particularly for those using metformin. These results should not be inferred to mean that physical activity is not necessary for individuals with type 2 diabetes who are using metformin as the benefits of physical activity are known to extend far beyond glycaemic control alone.^25^

There is literature suggesting that physical activity is associated with improvements in glucose control that is additive to the effects of metformin,^12^, ^13^, ^14^ or may even be associated with increased hepatic glucose production.^11^, ^15^ These studies are mainly acute effects of exercise on various measures of postprandial glucose control, or other measures of metabolism, and thus may not necessarily translate into chronic glycaemic control as reflected by HbA1c. Further, it is also important to note that the threshold for physical activity status used in the study was quite low (1 bout of MVPA or VigPA per month). Despite the low threshold, physical activity was still associated with more beneficial glycaemic profiles in those not using metformin. This reinforces the potential importance of even small amounts of physical activity, even if they are under the recommended amounts by current guidelines. It is important to highlight that even with the low threshold for activity status, only 22% of individuals reported engaging in vigorous PA at least once per month and 48% reported engaging in moderate‐to‐vigorous activity at least once per month. This represents a serious concern and suggests that physical activity promotion efforts are needed for individuals with type 2 diabetes.

The strengths and limitations of the current study warrant mention. This study used a large sample size of adults with type 2 diabetes who participated in NHANES (1999‐2018) surveys weighted to be representative of all non‐institutionalized citizens in the US Diabetes was defined using physiological measures of HbA1c, fasting glucose or glucose tolerance or the use of diabetes medications meaning that undiagnosed diabetes was also included in this sample. Though we adjusted for the use of other diabetes medications, there may be differences in the effectiveness between the types of medications taken, and nondiabetes medications may also have impacted glucose control. The data used in this study were cross‐sectional; therefore, no causation can be implied. All physical activities were self‐reported. Thus, individuals may have overestimated or underestimated their physical activity.^26^, ^27^, ^28^ However, error in self report would likely bias our observations to the null. Longitudinal assessment of physical activity using objective measures is needed to inform physical activity effects in preventing the complications of high glucose levels in type 2 diabetes patients.

These results suggest that physical activity may be associated with better glycaemic control only in individuals with type 2 diabetes who are not taking metformin. Metformin was associated with better glycaemic control profiles regardless of whether or not they were engaging in physical activity.

## CONFLICT OF INTEREST

All authors have no related conflicts of interest to declare.

## Data Availability

Data are publicly available at: https://wwwn.cdc.gov/nchs/nhanes/Default.aspx
